# Reviews for multimorbidity risk in people with inflammatory conditions: a qualitative study

**DOI:** 10.3399/BJGPO.2024.0011

**Published:** 2024-07-24

**Authors:** Lauren Gray, Laurna Bullock, Carolyn A Chew-Graham, Clare Jinks, Zoe Paskins, Samantha Hider

**Affiliations:** 1 Haywood Academic Rheumatology Centre, Midlands Partnership University Foundation Trust, Stoke-on-Trent, UK; 2 School of Medicine, Keele University, Keele, UK

**Keywords:** review consultations, multimorbidity, inflammatory arthritis, arthritis, rheumatoid, qualitative research

## Abstract

**Background:**

People with inflammatory rheumatological conditions (IRCs) are at high risk of developing other conditions including cardiovascular disease (CVD) and mood disorders.

**Aim:**

To explore perspectives of people with IRCs and healthcare practitioners (HCPs) on the content and delivery of a review consultation aimed at identification and management of multiple long-term conditions (mLTCs).

**Design & setting:**

Semi-structured interviews and focus groups with people with IRCs and HCPs in primary and secondary care.

**Method:**

People with IRCs participated in individual semi-structured interviews by telephone or online platform. HCPs (including primary and secondary care clinicians) participated in online focus groups. Data were transcribed verbatim and analysed using inductive thematic analysis.

**Results:**

Fifteen people with IRCs were interviewed; three focus groups with HCPs were conducted. The following two main themes were identified: reflecting on the value of review consultations; and what would a new review look like? Overall, people with IRCs and HCPs reflected that access to reviews is inequitable, leading to duplication of reviews and fragmentation in care. People with IRCs, at times, had difficulty conceptualising reviews, especially when discussing their future risk of conditions. People suggested that preparation before the healthcare review could align patient and HCP agendas as part of a flexible and person-centred discussion.

**Conclusion:**

Any review introduced for people with IRCs must move beyond a ‘tick-box’ exercise. To gain maximum value from a review, preparation from both patient and HCP may be required alongside a person-centred approach while ensuring they are targeted at people most likely to benefit.

## How this fits in

Current evidence shows that people with inflammatory rheumatological conditions (IRCs) are at high risk of developing other conditions including cardiovascular disease (CVD) and mood disorders. This qualitative study aimed to explore healthcare reviews to identify and manage risk of multiple long-term conditions (mLTCs) for people with IRCs. Our findings demonstrate the importance of person-centred healthcare reviews to minimise duplication and respond to patient need, ensuring that they are targeted at people most likely to benefit. And, to gain maximum value from a review, preparation from both patient and HCPs may be required alongside the person-centred approach.

## Introduction

Care for people with multimorbidity, or multiple long-term conditions (mLTCs), as is the preferred term,^1^ is a key challenge for the NHS.^2^

People with inflammatory rheumatological conditions (IRCs), such as rheumatoid arthritis (RA), axial spondyloarthritis (AS), and psoriatic arthritis (PsA), are more likely to develop other common conditions such as cardiovascular disease (CVD)^3,4^ and anxiety and/or depression,^4–6^ leading to increased morbidity, mortality, and worse outcomes from IRCs.

People with IRCs are more likely to have risk factors for poor health such as obesity, low levels of physical activity,^7,8^ and smoking,^9,10^ which can impact both on outcomes from the IRC and future mLTC risk.^10^ Addressing these risk factors early could potentially improve health outcomes. National Institute for Health and Care Excellence (NICE) guidance suggests that people with RA should be offered an annual review,^11^ although this was not offered for other IRCs. For people with RA this was offered within primary care as part of the Quality and Outcomes Framework (QOF), although this was retired as an indicator in 2023.^12^ Therefore, currently reviews are not incentivised and consequently not routine. Currently, annual reviews may occur in primary or secondary care although delivery is patchy, and it is not clear where responsibility for reviews lies.

Many people with IRCs will have other long-term conditions (LTCs) for which reviews are indicated (such as diabetes) although these reviews often focus on a single disease, rather than broader risks of mLTCs. These reviews are usually undertaken by practice nurses as part of the broader primary care team. We previously developed and tested a face-to-face nurse-led review (the INCLUDE review) in primary care that addressed CVD risk, bone health and fracture risk, and anxiety and depression in people with IRCs.^13^ This was shown to be feasible, deliverable, and was associated with changes in management (including increased prescribing especially around CVD and osteoporosis) and was acceptable to patients.^14,15^ We highlighted the challenges of multiple healthcare reviews for people with mLTCs and issues in deciding what problems should be prioritised.^15^

Since the INCLUDE study, the context for healthcare reviews for people with LTCs has changed considerably. First, restrictions during the COVID-19 pandemic led to rapid adoption of remotely delivered health care and more digitally enabled care. Some elements of ‘digital first’ are a key part of the *NHS Long Term Plan*.^16^ There remain concerns around the potential transactional nature of remote consultations, how to optimise them to help manage people with mLTCs rather than acute problems, and how to best ensure equity of access.^17^ While uptake for NHS health checks for CVD is broadly representative of the UK population, there remains considerable variation, with recent recommendations suggesting that reviews should be more person-centred and targeted more at those at highest risk, taking account of health inequalities.^18^

Given the changing context of healthcare reviews for people with IRCs, we sought to explore patient and practitioner perspectives on healthcare reviews to consider current practice and how reviews could be optimised for people with IRCs who are at high risk of future mLTCs.

### Aim

The aims of the study were as follows: (a) to explore perspectives of people with IRCs about the content and delivery of review consultations to identify and manage risk of developing other LTCs; and (b) to explore practitioner perspectives regarding current practice, feasibility, and acceptability of healthcare reviews for people at high risk of future mLTCs.

## Method

We conducted semi-structured interviews with people with IRCs and focus groups with healthcare practitioners (HCPs) involved in the design or delivery of healthcare reviews. Focus groups provided an interactional dynamic between HCPs in different roles and ways of practice, which enriched the data. Whereas, people with IRCs were interviewed individually as it was more appropriate when discussing potentially sensitive subjects (for example, mood and weight).

People with IRCs were recruited from (a) people who had taken part in previous research studies locally and provided consent to future contact; (b) local research user groups, patient-facing organisations, and networks; or (c) X (formerly known as Twitter). HCPs were recruited via primary and secondary care networks and cascaded via the National Institute for Health and Care Research (NIHR) Clinical Research Network, integrated care boards, and X. Owing to the heterogeneity in HCPs who deliver reviews, recruitment included both primary and secondary care roles to capture the broadest range of perspectives.

Topic guides were developed with input from the patient and public involvement (PPI) group and updated iteratively in line with data generation and analysis. Individual interviews were conducted either by telephone or Microsoft Teams and focus groups were conducted using Microsoft Teams.

Data collection and analysis were undertaken between May and October 2022 by LG (qualitative PhD researcher) and LB (experienced qualitative researcher). Data were audio-recorded, transcribed verbatim, coded (LG and LB), and managed with NVivo software (version 12). Line-by-line coding and inductive thematic analysis^19,20^ was undertaken (LG and LB). Both LB and LG are non-clinical researchers, potentially affecting data collection and analysis. Reflexive practices (for example, a reflexive diary) allowed decisions to be audited throughout the research process. Data collection and analysis were regularly discussed with the wider research team that included primary and secondary care clinicians, to discuss data interpretations, enhancing credibility and trustworthiness,^21^ and key themes agreed using the principles of constant comparison.^22,23^ The final sample size was informed by information power; a pragmatic judgement based on a number of items including aims, specificity, theory, dialogue, and analysis.^24^ The sample size was kept under review through concurrent data collection and analysis, and stopped when no more unique codes were identified.

### Patient and public involvement

PPI members, with lived experience of IRCs, were recruited to a patient advisory group (PAG). One online 2-hour workshop, attended by four members (three female and one male), was convened during the early stages of data collection to ensure that PPI perspectives supported data interpretation. To do this, PAG members were presented with anonymised data extracts. Members discussed their interpretations and understandings of the data and what this means for the future of healthcare reviews for people with IRCs. Throughout all discussions, PAG members reiterated the importance of IRC reviews being person-centred in content and delivery to adequately address patient needs.

## Results

### Demographics

Fifteen interviews took place with people with an IRC (RA = 10, AS = 3, PsA = 1, systemic lupus erythematosus [SLE] = 1) and included eight males and seven females with a mean age of 60.4 (range 49–75) years (Table 1).

**Table 1.** Demographic details of interview participants with IRCs

Characteristic	People with IRCs (*n* = 15)	
Sex	Females	8
	Males	7
Age	40–49	1
	50–59	4
	60–69	4
	70–79	6
Condition	RA	10
	PsA	1
	AS	3
	SLE	1
Multiple LTCs		14/15 participants

AS = axial spondylarthritis. IRC = inflammatory rheumatic condition. LTCs = long-term conditions. PsA = psoriatic arthritis. RA = rheumatoid arthritis. SLE = systemic lupus erythematosus.

Three focus groups (*n* = 4; *n* = 6; *n* = 4) were conducted with HCPs involved in the care of people with IRCs, including a range of different professional backgrounds (see Table 2).

**Table 2.** Details of healthcare professionals participating in focus groups

Heath professional participants (*n* = 14)	
Sex female = 11 participants	
HCP1 HCP2 HCP3 HCP4	Dietician Dietician Occupational therapist Consultant rheumatologist
HCP5 HCP6 HCP7 HCP8 HCP9 HCP10	Nurse Physiotherapy practitioner Physiotherapy practitioner MSK healthcare assistant Public health Public health
HCP11 HCP12 HCP13 HCP14	Advanced nurse practitioner GP Advanced nurse practitioner GP

HCP = healthcare practitioner. MSK = musculoskeletal.

**Figure 1.**
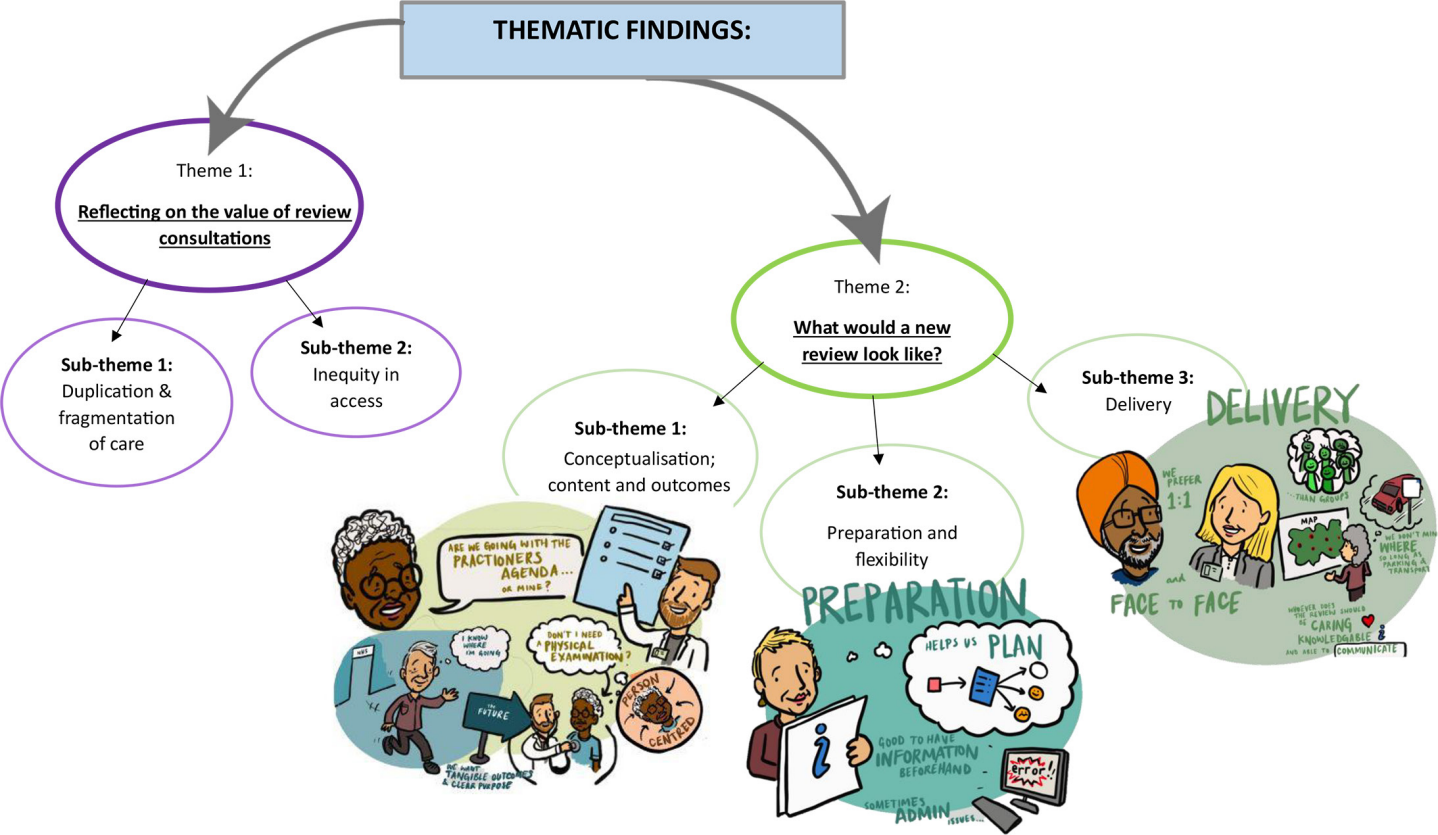
A summary of the themes and sub-themes

The following two main themes were identified: reflecting on the value of review consultations; and what would a new review look like? For the first theme, the following two sub-themes were identified: duplication and fragmentation; and inequity in access. When considering the design of a new review, the following three sub-themes were identified: conceptualisation of the review; preparation and flexibility; and delivery. Details of the themes are presented along with the individual sub-themes using illustrative quotations. Unique identifiers for people with IRCs interviews (P) include their participant number and for HCP focus group number (FG). An overview of themes and sub-themes is summarised pictorially in Figure 1.

### Reflecting on the value of review consultations

#### Duplication and fragmentation of care

HCPs suggested that QOF meant that people with mLTCs may be invited to multiple reviews about individual conditions, with some patients even receiving duplicate reviews:

*‘I think what the problem is with QOF is that it deals with individual conditions and risk factors for those and the next step in my view should be that we need to start to align some of that around multimorbidity.’* (FG03, P14)

People with IRCs reported the number of reviews added to their treatment burden and that reviews tended to have a single disease agenda with little room to discuss the wider context and/or address their own agenda:

*‘You can feel like you’re just going from one review to the next and back round again.’ (P14*)

*‘You don't get treated as a patient as a whole patient, you get treated for one bit or you get treated for another bit or another bit*.’ (P01)

HCPs described being under increasing pressure to meet the requirements of the QOF, and with time-limited appointments, there was little space for the patient agenda or exploration of patient concerns:

*‘There’s a lot of pressure on completing those tick boxes. And if you forget one, you don't get the points and you don't get the payment.*’ (FG03, P11)

Poor integration and challenges around communication between primary and secondary care providers lead to further challenges for practitioners and patients:

*‘How can we join up the patient's care and ask all these different people to be involved in that patient’s care when actually we can't even see their record from one building to the next so not having the systems there to support the clinicians.*’ (FG02 P07)

*‘The NHS does not talk to each other … They are separate silos.*’ (P01)

Consequently, this can lead to some patients going without a review, while others receive duplicate reviews:

*‘And if they are attending those reviews* [with secondary care] *we don't tend put them in with the GP for another review*.’ (FG03, P11)

This fragmentation of care may impact on person-centredness as patients reported feeling they are not being viewed as a ‘whole person’. People who had experienced LTC reviews suggested that reviews are just a ‘tick-box exercise’:

*‘I feel like it’s just a box that they've got a tick. They do it* [the review] *and then I'm out of there without feeling like I've participated really*.’ (P07)

HCPs described the challenges aligning patient and practitioner agendas in one appointment to create a more holistic approach, owing to time constraints:

*‘The patient’s priorities are very different to the clinician's and what’s important to them can be worlds apart really*.’ (FG01, P03)

*‘We'd all love to fully understand all of our patients and you know, concerns and expectations but in the real world when you've got 10 minutes … it really doesn't always play out that way.*’ (FG03, P14)

#### Inequity in access

HCPs reported a gap in providing care that helps detect and prevent health problems to those who need it most. HCPs suggested that people who would benefit most from a risk assessment review, those most likely to be at risk of mLTCs, may also find reviews more difficult to access. Both people with IRCs and practitioners suggested that patients with greater needs owing to limited health literacy, poor digital access, or poor mobility would find it more difficult to access reviews.

*‘Accessibility of those services is disproportionately difficult for people who have the greatest health needs.’* (FG02, P10)

*‘You can't always get on this particular booking platform that’s on there* [online] *which makes it a bit difficult.*’ (P16)

HCPs highlighted the need for reviews to target underserved communities, as they are more likely to experience higher disease burden yet may access care less frequently:

*‘I think one of the challenges that exists fundamentally is that most of the diseases that we'll be talking about will have a greater impact and there'll be a higher disease burden in the communities that have got higher levels of socioeconomic deprivation.*’ (FG02, P10)

However, inequities in care commissioning can mean that those who are most in need receive less care:

*‘You know it is a postcode lottery for some of these people*.’ (FG02, P07)

### What would a new review look like?

In considering what would a new review look like, we identified the following three sub-themes: conceptualising content; need for preparation and flexibility; and delivery. These are summarised pictorially in Figure 1.

#### Conceptualisation; content and outcomes

People with IRCs struggled to conceptualise what an IRC annual review would include. At the time of interview one person with an IRC had received invitation from their GP for their RA review but had no insight into what this would involve:

'*I’ve had a text message — It says on the text your annual rheumatoid review. That’s a first. Never had one before so. What that entails I do not know until I’ve been.*’ (P16)

The idea of having a ‘RA review’ yet talking about other conditions, such as CVD or osteoporosis, did not align for some people with IRCs as this was not something they expected. Given that people with IRCs conceptualise their review to be a holistic ‘whole-person’ approach, many people suggested that a physical examination would be an important part of such a review:

*‘I think they* [the review] *should be in depth, including a physical examination.*’ (P16)

Conversations surrounding mood and lifestyle were accepted and generally areas that patients wanted to be explored within such a review:

*‘It'd be nice to chat about it, I think. If there’s anything any way that you could be doing things differently that might help.*’ (P02)

People with LTCs described a range of attitudes regarding whether they would be motivated to attend a review focused on their risk of potential future conditions. Some perceived increasing awareness of their personal risk of other conditions as negative:

*‘Having someone talking about the possibilities of related conditions would simply serve to make me feel more anxious.’* (P12)

Whereas others perceived it more positively:

*‘Forewarned is forearmed, if you know what’s coming you can do something about it*.’ (P10)

For a review to be perceived as valuable, people with IRCs considered it important to have clear understanding of the review purpose and outcomes:

*‘If nothing is going to come of it (…) not going to follow-up on something that I’ve said then what is the point of ringing me because all you have done is taken 10 minutes out of my day.’* (P10)

#### Preparation and flexibility

People with IRCs and practitioners suggested that preparation resources (for example, written information sent out to a patient before the review) may be beneficial. This could provide an opportunity to align patient and HCP expectations and agendas, helping patients identify their priorities for discussion. To accommodate this person-centred approach, the review must remain flexible and responsive to the patient agenda. One HCP reflected that this approach had potential to increase patient engagement with healthcare reviews and improve health outcomes:

*‘We just invite patients to a chronic disease review and they turn up thinking it’s something different. So I think outlining what our aims are for the purpose of the appointment helps align it with patient’s expectations of the review. I think that’s something I’ll feedback to our team (…) because that does sound like it might save us some of the difficulties, we have in getting patients to attend and engage.*’ (FG03, P12)

Preparation materials were considered of high importance but would only be meaningful if they were accessible to all. One person with an IRC spoke about how family members would differ in their ability to access preparation materials owing to reading and writing comprehension:

*‘It’s* [RA] *still seen as an elderly disease so whether they are able to do this* [preparation]*. My mum would, but my mother-in-law, probably not. So would that mean she got less care because they didn't do it? So, I don't know how you would encompass everybody.’* (P13)

The additional administrative requirements for this to work efficiently was also highlighted as a consideration:

*‘It’s fine sending things out and getting things back, but somebody has to look at it and code it and put it on, so it’s lots of admin.*’ (FG03, P11)

#### Delivery

Participants discussed the delivery of the review, and where it should take place. All people with lRCs suggested that they would prefer to have a face-to-face review rather than a remote consultation. However, patients acknowledged that the delivery of a review needs to consider patient preference and needs. This is particularly the case for patients who may have barriers to communication or impaired mobility:

*‘Some people find it hard to get to whether they’re going to go, so they might prefer over the phone. But I don't. I prefer to go and sit and talk with somebody.*’ (P16)

Patients suggested that the review could take place in primary or secondary care, as long as practicalities were considered such as travel and parking:

*‘It’s just I suppose where it was and if I can get to there you know transport and how far away it was, how near.*’ (P11)

Practitioners from both primary and secondary care suggested that the review should take place in primary care, offering more ‘holistic’ consultations, compared with specialist care that focused on the index IRC and its treatment:

*‘Their attitude is that the rheumatologist is there to make the decision about treatment.*’ (FG03, P12)

*‘Lots of that* [review content] *is general health stuff. And I think the more that we* [secondary care] *take on of that, there is a risk that the GPs will view that as "oh, they're under rheumatology and therefore we better not do anything".’* (FG01, P04)

People with IRCs and HCPs reflected that GPs were ideal to undertake holistic reviews. However, when reflecting on existing pressures and rising demand on GPs, practitioners also discussed utilising broader primary care roles. These roles (lifestyle coach, wellbeing practitioner and so on.) were suggested to be helpful supporting people at risk of mLTCs, providing them with the time they need:

*‘Our lifestyle coach was brilliant. He would see people with difficulties dealing with their comorbidities on a regular basis and he had the time to follow them up (...) He had really good results like that because they engaged with him.*’ (FG03, P11)

However, people with IRCs and HCPs stressed that, regardless of the HCP role, appropriate skills and expertise are needed to be able to cover all areas sufficiently. Specifically, knowledge of the IRC was deemed important:

*‘It’s got to be someone who’s got some inside knowledge of the condition and how it affects people in general. Obviously, they’ve got to have a good insight and good knowledge of how to advance care.’* (P06)

*‘It’s about the expertise, isn't it? That you need to be able to review that patient correctly because you know you need certain competencies and expertise to be able to manage the condition.’* (FG02 P09)

## Discussion

### Summary

This qualitative study explored perspectives on the content and delivery of healthcare reviews to identify and manage risk of mLTCs for people with IRCs. Patients and practitioners reflected on the importance, yet difficulty, of providing person-centred care by aligning agendas and targeting these reviews to people who are most at need.

Among people with IRCs there is confusion about the content and purpose of mLTC healthcare reviews, leading to challenges aligning practitioner and patient agendas in the time allotted. Giving patients time to prepare with accessible materials sent out before the review was considered an important factor in helping address this confusion and ensuring flexibility in the review to accommodate patient agendas.

### Strengths and limitations

To our knowledge this is the first study that has explored patient and practitioner perspectives on reviews to assess mLTC risk for those with IRCs. Strengths of the study include the embedded PPI involvement, which informed the study improving the relevance and credibility of our findings. Topic guides used for data collection provided a broad range of discussion elements adapted to fit individual circumstances, which were developed iteratively alongside data collection. Practitioners recruited for focus groups came from a variety of primary and secondary backgrounds enabling a broad perspective to be gathered. Weaknesses include that among the interviews with people with IRCs although we recruited a broad spectrum of age, sex, and inflammatory conditions, there was limited ethnic diversity.

### Comparison with existing literature

People with mLTCs are particularly challenged in navigating a fragmented care system that is designed to treat diseases, not people.^25^ This is reflected in our patient population where those living with an IRC and other conditions reported they were often attending multiple appointments with separate services, resulting in treatment burden.^26,27^

The inverse care law^28^ describes how people who most need health care are the least likely to receive it. In particular, the challenge surrounds enabling people with mLTCs to live well in the community by providing care according to their needs, which will subsequently reduce pressure on emergency services, reduce fragmentation of care, improve health, and narrow health inequalities.^29^ A review of the NHS Health Check programme highlighted these need to be better targeted at those most likely to benefit^18^ and take account of socioeconomic inequalities that may mean people are less likely to seek care^30^ or face barriers to doing so.^31^ COVID-19 has perpetuated the widening of health inequalities, which is reflected in NHS priorities (for example, the ‘eight urgent actions’).^32^ This includes accelerating preventive programmes that proactively engage those at risk of poor health outcomes.^32^

Lack of person-centred care leads many people with IRCs to describe reviews as a ‘tick-box exercise’. People with mLTCs want individualised care,^33^ which is integrated and well-coordinated and delivered with an understanding of the context of their lives, respecting their goals, preferences, and values.^25^ However, HCPs in this study describe challenges aligning patients and practitioner agendas, to give the patients the holistic approach that they want. Reasons for this include pressures from QOF targets and time constraints, highlighted in international studies.^34^ Evidence suggests that longer consultations are a cost-effective way of increasing patient quality of life^35^ and result in more preventive health services,^36^ allowing the space and time needed to assess individualised risk.

People with IRCs had a range of views on whether they would be motivated to attend a review focused on their risk of potential future conditions. Previous research found some patients valued personalised risk information and acknowledged several potential benefits, including helping understand their diagnosis and help with informed decision making.^37^ Patients in previous research also preferred disclosure of risk information^38^ even if it did not influence their behaviour, as a means of simply being informed.^39^ In our study other participants were concerned about receiving risk information owing to the uncertainty around risk or reported that this could increase anxiety and negatively impact mental health.^38^ Thus, not all patients will want or benefit from a review aimed at assessing their future risk of conditions. People should be offered shared decision making as to the extent of risk assessment with universal precautions for health literacy.^40,41^

For a review to be perceived as valuable, people with IRCs in this study considered it important to have a clear purpose and understanding of the review. Many struggled to conceptualise a healthcare review looking at their future risks, rather than addressing their IRC specifically as this did not align with their expectations, which emphasised expectations around physical examination. Bowling *et al* ^42^ highlighted patient expectations of a review were that something would be done (for example, a referral) or offered (for example, receiving lifestyle advice). Similar expectations are seen in this study, with patients being unclear about the purpose and tangible outcomes. Our study highlights tension around effective review delivery, as while generally the review was considered best delivered in primary care, people wanted the reviewer to understand and address their IRC specifically.

Preparation was considered key to align patient and practitioner agendas and expectations, but that this would be adaptable to patient preference and needs. The 3D study^43^ showed that patient preparation helped identify priorities and facilitate individualised consultations and communication.^44,45^ However, a Cochrane review^46^ suggested pre-consultation preparation materials were of limited benefit. Although this was published in 2007 and, given the increase in remote healthcare delivery post-COVID-19, further research into acceptable patient preparation materials is warranted.

The Nuffield Trust suggests using a broader practitioner skill mix supports access and continuity in primary care.^47^ Our findings support that there is opportunity to utilise new wider primary care roles to provide valuable holistic care, while reducing the pressure on GPs but knowledge of IRCs may be a training need.

### Implications for research and practice

These findings can help HCPs, policymakers, and providers further their understanding on preventing mLTCs for people at high risk, moving beyond a one-size-fits-all to a more person-centred approach, where reviews address more than one LTC, although delivering this in practice may be challenging. Using preparation resources and ensuring flexibility in the review may help align agendas.

However, for this to be implementable and effective, there is a need for increased use of shared care arrangements between services, while considering challenges including poor IT integration, which can contribute to disconnect between services, inequity, and duplication. Using newer roles, such as health coaches and social prescribers, could support these approaches, although knowledge around IRCs may be a training need.

These findings highlight the need to adapt healthcare reviews to the rising prevalence of mLTCs to ensure such reviews are optimised and taken up by people most likely to benefit.

## Data

The dataset relied on in this article is available from the corresponding author on reasonable request.
